# Investigation of Algerian *Crataegus monogyna Jacq* Phenolic Compounds (Using LC-ESI-MS/MS Analysis, Antioxidant Activity, and Enzyme Inhibition) and Their Potential Implications for Food and Nutraceutical Applications

**DOI:** 10.3390/antiox13111350

**Published:** 2024-11-04

**Authors:** Sabrina Goudjil, Samira Boussekine, Sarra Goudjil, Hanane Goudjil, Mustafa Abdullah Yilmaz, Mohammad Shamsul Ola, Ahmad Ali, Oguz Cakir

**Affiliations:** 1 Laboratory of Bioactive Molecules and Applications, Department of Applied Biology, Faculty of Exact Sciences and Natural and Life Sciences, Echahid Cheikh Larbi Tebessi University, Tebessa 12000, Algeria; 2 Laboratory of Didactics of Physical and Chemical Sciences and Applications, Assia Djebar Higher Normal School of Constantine, Constantine 25000, Algeria; goudjil.sarra@ensc.dz; 3 Department of Mining Engineering, Mining Operations, Faculty of Engineering, Karadeniz Technical University, Trabzon 61080, Turkiye; 422595@ogr.ktu.edu.tr; 4 Department of Analytical Chemistry, Faculty of Pharmacy, Dicle University, Diyarbakir 21280,Turkiye; mabdullah.yilmaz@dicle.edu.tr (M.A.Y.); oguz.cakir@dicle.edu.tr (O.C.); 5 Dicle University Science and Technology Research and Application Center, Diyarbakir 21280, Turkiye; 6 Department of Biochemistry, Faculty of Science, King Saud University, Riyadh 11415, Saudi Arabia; mola@ksu.edu.sa; 7 Department of Life Sciences, University of Mumbai, Vidyanagari, Mumbai 400098, India; ahmadali@mu.ac.in

**Keywords:** *Crataegus monogyna Jacq*, LC-ESI-MS/MS, butanolic fraction, polyphenol, hesperidin, urease, AChE, *α*-amylase, antioxidant activity

## Abstract

Investigations into the phenolic constituents of the butanolic fraction of *Crataegus monogyna* were optimized using LC-ESI-MS/MS analysis, identifying and quantifying at least 23 fingerprint phytochemical compounds. The major phenolic compounds were epicatechin (99.916 ± 2.208 mg/g), isoquercetrin (53.31 ± 1.172 mg/g), chlorogenic acid (47.457 ± 1.010 mg/g), quinic acid (37.819 ± 1.406 mg/g), rutin (29.98 ± 0.740 mg/g), hesperidin (5.296 ± 0.177 mg/g, detected for the first time in the *C. monogyna* species), astragalin (1.774 ± 0.020 mg/g), and nicotiflorin (1.482 ± 0.016 mg/g). The antioxidant properties of the lyophilized butanolic fraction were evaluated using DPPH, GOR, ABTS, CUPRAC, and reducing power assays, all of which demonstrated that there was strong activity. Additionally, the neuroprotective effect was evaluated in vitro, showing a potent inhibitory effect on acetylcholinesterase (AChE) with an IC50 of 43.65 ± 2.10 µg/mL. The antidiabetic effect was investigated through *α*-amylase inhibition (IC50 = 91.19 ± 0.10 µg/mL), showing high inhibitory activity. In addition, the butanolic extract exhibited significant urease inhibition with an IC50 of 26.36 ± 0.05 µg/mL. These results suggest that Algerian *C. monogyna* has potential as a therapeutic agent for managing diabetes complications and as a natural source of AChE inhibitors, making it a promising subject for the treatment of urease-related conditions. Its high concentrations of natural antioxidants, such as epicatechin, isoquercetrin, chlorogenic acid, quinic acid, rutin, hesperidin, and astragalin, make it suitable for integration into medicine, pharmaceuticals, cosmetics, and the food sector.

## 1. Introduction

Interest in traditional medicine is constantly increasing. The growing interest in natural products has prompted extensive scientific research to gain a deeper understanding of medicinal plants and herbal formulations due to their affordability, accessibility, safety, and lower toxicity. They are considered a rich and efficient source of active biological compounds for the development of novel products used for taste, aroma, color, and additives in foods [1], as well as in the cosmetic and pharmaceutical industries and for treating various human diseases [2,3,4]. Their composition in bioactive compounds is generally responsible for their biological activity [5]. Secondary bioactive metabolites like alkaloids, procyanidins, flavonoids, and polyphenols found in plants exhibit antioxidant effects and are implicated in the prevention and treatment of various diseases linked to oxidative stress, such as cancer, diabetes, inflammatory conditions, and neurodegenerative diseases [6,7]. One of these interesting traditional medicinal herbs is hawthorn (*Crataegus* spp.) [8]. Hawthorn is an endemic member of the *Rosaceae* family [9]. The genus *Crataegus* comprises approximately 300 species and is widely distributed in North Africa, Europe, West Asia, and North America [10]. One of these species is *Crataegus monogyna Jacq* [7]. Its leaves, flowers, and berries are commonly used in folk medicine [2] to treat chronic and congestive heart failure [11], high blood pressure, hypertension, arrhythmia, irregular heartbeat, and various digestive ailments. Additionally, it is employed as a geriatric and anti-arteriosclerosis remedy, providing protection against angina, chest pain, and arterial hardening [12]. Moreover, it serves as a sedative, diuretic, anti-inflammatory, and cardiotonic agent [13], offering protection against insomnia and anxiety [14]. It is classified as a “cardiotonic” herb [12]. In the European tradition, *Crataegus* (leaves and flowers) have long been used as astringent and anti-atherosclerotic agents [2]. In traditional Chinese medicine, they are utilized to eliminate blood stasis, enhance blood circulation, and to treat diarrhea, indigestion, hyperlipidemia, abdominal pain, and hypertension [11]. These pharmacological properties are attributed to the beneficial effects of active phenolic molecules in *C. monogyna Jacq*, which regulate various biological metabolisms [15]. *C. monogyna Jacq* fruits, leaves, and flowers contain a variety of chemical compounds. For instance, flavonoids are present in varying concentrations (0.1–1% in fruits and 1–2% in leaves and flowers), including hyperoside, rutin, vitexin, acetylvitexin-2″-O-rhamnoside, and vitexin-2″-O-rhamnoside [16]. Oligomeric proanthocyanidins (OPCs, 1–3% in fruits or leaves with flowers) are composed of chains of flavan-3-ol units [10]. Organic acids (2–6%) [17], triterpene acids (0.5–1.4% in fruits: oleanolic and ursolic acids) [18], phenolic acids (chlorogenic and caffeic acids), saponins, some cardioactive amines, and sterols [19] are also found. Generally, flavonoid glycosides, bioflavonoids with oligomeric procyanidins, phenolic acids, flavone, and various types of flavonoids are the primary molecules responsible for the biological activities of *Crataegus* species [11]. Due to its significant phenolic content, *C. monogyna Jacq* is an excellent source of natural antioxidant compounds, including chlorogenic acid, hyperoside, rutin, isoquercetin, quercetin, epicatechin, and protocatechuic acids [19].

Furthermore, to extend and ensure the optimum shelf life of food products, several studies have evaluated natural antioxidants and antimicrobials, including plant extracts and essential oils, as alternatives to synthetic antioxidants in the food industry. Currently, natural antioxidants are not widely used due to their high price and limited sources. However, identifying new sources of safe and cost-effective naturally occurring antioxidants could prove beneficial for both the food industry and pharmaceuticals, with the aim of replacing synthetic antioxidants. The advantages of their use are numerous [19]. Hawthorn polyphenols, known for being natural, healthy, and effective, and for having low-toxicity, are frequently employed in the food industry, medical treatments, and health products [1,8]. The butanolic extract of *C. monogyna* has gained attention for its rich phenolic content and therapeutic potential, particularly its antioxidant properties. The literature provides limited information regarding this subject. The hypotheses suggest that the phenolic compounds in this extract can prevent oxidation, thereby extending the shelf life of food products. This section summarizes recent research advances and highlights the novel contributions of this study, which focus on the biological activities of phenolic biomolecules derived from the lyophilized butanolic fraction of *C. monogyna* (leaves, fruits, and flowers). In evaluating the Algerian natural surface environment for developing new natural bioactive molecules that contribute to therapeutic effects and functional metabolites in herbal food, cosmetics, food additives, and pharmaceuticals, this study represents the first comprehensive investigation into the total phenolic content (phenols, flavonoids, and flavonols), antioxidant potential (DPPH, ABTS, GOR, CUPRAC, and reducing power), enzyme inhibitory activities (acetylcholinesterase and urease), and antidiabetic properties (alpha-amylase) of this extract. The phenolic compounds in this fraction were identified and quantified using the developed liquid chromatography–tandem mass spectrometry (LC-ESI-MS/MS).

## 2. Materials and Methods

### 2.1. Extraction Procedure of Butanolic Fraction

The leaves, fruits, and flowers of *Crataegus monogyna* were collected from the Forest of Oued Souffay in Aïn Defla, Algeria (Latitude: 36°14′5.47″ and Longitude: 2°13′39.36″) during the flowering period at the end of March 2020. They were identified by Prof. Heoun from the Faculty of Biology, Tebessa University, Algeria. Subsequently, the leaves, fruits, and flowers were cleaned, air-dried in the dark at ambient temperature (25 °C), and stored. Extraction of phenolic compounds was performed using the method outlined by Markham (1982) [20], with modifications adapted from Bruneton (1993) [21]. Four hundred grams of a mixture, consisting of approximately 130 g each of flowers, fruits, and leaves of *C. monogyna*, were extracted three times with 4 L of methanol–water (80:20 *v*/*v*) for 24 h. The residue was evaporated and concentrated. The crude extract was suspended in 300 mL of hot distilled water and cooled overnight to separate chlorophyll. The resulting filtrate was successively extracted with petroleum ether (4 × 200 mL), diethyl ether (4 × 200 mL), ethyl acetate (4 × 200 mL), and n-butanol (4 × 200 mL). The butanolic extract was used in our study; it was evaporated, frozen, and lyophilized to obtain 14 g of lyophilized powder from the *C. monogyna* butanolic fraction.

### 2.2. Reagents and Standards

#### Reagents for LC-ESI-MS/MS Analysis

##### Preparation of Standards Solutions

In order to establish the analytical approach, 53 naturally occurring phenolic compounds and 3 isotope-labeled phenolic compounds (internal standards) were utilized as authentic standards (Figure S1, Supplementary Material). The 56 standard compounds, including ISs, were dissolved in methanol to provide 1000 mg/L main stock solutions. The compounds were acquired in their solid form. Exceptionally, epicatechin and epigallocatechin standard main stock solutions were prepared at a concentration of 500 mg/L. In addition, 53 phenolic standards were generated as intermediate stock solutions to make the procedure easier. Isotope-labeled internal standards for the measurement of non-flavonoid substances, flavonoid glycosides, and flavonoid aglycones were ferulic acid D3, rutin D3, and quercetin D3, respectively. To create the calibration curve, eight levels were established, each having a combination of three ISs and fifty-three phenolic standards. Each calibration level and plant sample was spiked with 20 mg/L of ferulic acid D3, 1 mg/L of rutin D3, and 5 mg/L of quercetin D3 [22].

### 2.3. Mass Spectrometer and Chromatography Conditions LC-ESI-MS/MS

Quantitative analysis of 56 phenolic compounds was performed using a Nexera Shimadzu ultra-high-performance liquid chromatography (UHPLC) model coupled with a tandem mass spectrometer system. The reversed-phase UHPLC was equipped with a SIL-30AC auto-sampler, a CTO-10ASvp column oven, LC-30AD binary pumps, and a DGU-20A3R degasser. To achieve optimum separation of 56 phenolic compounds and to overcome suppression effects, the chromatographic conditions were optimized using a reversed-phase Agilent Poroshell 120 EC-C18 model (150 mm × 2.1 mm, 2.7 µm) analytical column. The column temperature was set at 40 °C. The mobile phase consisted of eluent A (water + 5 mM ammonium formate + 0.1% formic acid) and eluent B (methanol + 5 mM ammonium formate + 0.1% formic acid) with a linear elution gradient: 20–100% B (0–25 min), 100% B (25–35 min), and 20% B (35–45 min). The flow rate of the mobile phase solvent was maintained at 0.5 mL/min, and the injection volume was set at 5 µL. Mass spectrometer analysis was performed using a Shimadzu LCMS-8040 tandem mass spectrometer equipped with an electrospray ionization (ESI) source operating in both positive and negative ion modes. LC-ESI-MS/MS data were monitored and processed using Shimadzu LabSolutions LC-MS software (version 5.81). For the quantification of phytochemical compounds, the multiple reaction monitoring (MRM) mode was used. The MRM method was optimized to selectively detect and quantify phytochemicals by screening specified precursor-to-fragment ion transitions. To generate optimal phytochemical fragmentation and maximal transmission of the desired product ions, the collision energies (CE) were optimized.

The mass spectrometer system conditions were examined as follows: DL temperature, 250 °C; interface temperature, 350 °C; drying gas (N2) flow, 15 L/min; heat block temperature, 400 °C; and nebulizing gas (N2) flow, 3 L/min [22].

### 2.4. Quantification of Total Phenolic Bioactive Substances

#### 2.4.1. Total Phenolic Content (TPC)

The total phenolic content of the n-butanolic fraction was determined using a modified form of Folin–Ciocalteu method [23]. The total phenolic content was expressed as gallic acid equivalents (GAEs) micrograms per milligram of extract.

#### 2.4.2. Total Flavonoids Content (TFC)

A modified method was used to determine the total flavonoid content of the n-butanol fraction [24]. The result was expressed in micrograms of quercetin equivalent per milligram of extract (μg QE/mg).

#### 2.4.3. Total Flavonols Content (TFL)

The total flavonols content of the n-butanolic fraction was determined using the method described by [25]. The total flavonol content was expressed in micrograms of quercetin equivalent per milligram of extract (µg QE/mg).

### 2.5. Antioxidant Activity

#### 2.5.1. DPPH Free Radical Scavenging Assay

1,1-Diphenyl-2-picrylhydrazyl (DPPH) radical scavenging activity of the n-butanol fraction was measured using a modified version of an existing method [26]. The concentration causing 50% inhibition (IC50) was calculated and compared with antioxidant standards (BHA, BHT, Trolox, and ascorbic acid).

#### 2.5.2. ABTS Cation Radical Assay

The free radical scavenging test of ABTS was evaluated using a slightly modified method of [27]. Butylated hydroxytoluene (BHT) was used as a positive control. The results are presented as 50% inhibitory concentration (IC50).

#### 2.5.3. GOR Scavenging Assay

Galvinoxyl free radical (GOR) scavenging activity was determined using the method described by [28].

#### 2.5.4. Cuprac Ion Reducing Antioxidant Capacity (CUPRAC)

The Cupric ion reducing antioxidant capacity (CUPRAC) of the extracts was determined using a modified method previously described by [29]. The results are given as absorbance and A0.5 (μg/mL), which correspond with the concentration, indicating 50% absorbance intensity, and are compared with those of the standards BHA and BHT.

#### 2.5.5. Reducing Power Assay

The reducing power was measured with slight modifications, according to the method described by [30]. Ascorbic acid, tannic acid, *α*-tocopherol, BHA, and BHT were used as standards. The results are presented as A0.5 (μg/mL), corresponding to the concentration and indicating 50% absorbance intensity.

### 2.6. Enzymatic Inhibitory Assays

#### 2.6.1. Acetylcholinesterase Inhibitory Activity (AChE)

The acetylcholinesterase inhibitory activity of the butanolic fraction of *C. monogyna* species was evaluated using the spectrophotometric method [31]. Acetylthiocholine iodide (AChI) was used as the substrate for enzyme activity studies, and galanthamine served as the standard compound. The results are expressed as 50% inhibition concentration (IC50).

#### 2.6.2. Amylase Inhibitory Activity

The amylase inhibitor activity of the butanolic extract was tested based on the method previously reported by Behvar et al. (2018). Acarbose was used as a standard *α*-amylase enzyme inhibitor [32].

#### 2.6.3. Urease Inhibition Activity

The Urease inhibitory activity was determined by measuring ammonia production using the indophenol method [33]. Thiourea was used as the standard inhibitor.

### 2.7. Statistical Analysis

All data results were expressed as mean values  ±  standard deviation (SD) of triplicate. The mean values were analyzed by Student’s *t*-test and one-way ANOVA followed by Tukey’s test using GraphPad Prism V: 8.00. Differences between means of parameters were considered significant, with *p* < 0.05. Correlation analyses were performed using a two-tailed Pearson’s correlation coefficient (ρ) test, also performed with GraphPad Prism V: 8.00.

## 3. Results and Discussion

### 3.1. Quantification of Total Phenolic Bioactive Substances

The growing recognition of the value of endemic and consumed plants has enhanced their application in the fields in pharmaceuticals, cosmetics, and food production. As demand for natural products rises, driven by the need to replace synthetic products with undesirable side effects, researchers are turning to nature for safer and more effective alternatives. Among these natural products, natural antioxidants play a crucial role in enhancing food and industry products by serving as natural additives, replacing synthetic chemical antioxidants. *C. monogyna Jacq*, commonly known as the common hawthorn, is a medicinal plant endemic to Algeria. It has a noteworthy chemical composition, being particularly rich in phenolic substances that offer valuable natural antioxidants. This plant is extensively utilized in traditional medicine for treating a range of conditions, including diabetes, cancer, and heart diseases [34].

The polyphenols in *C. monogyna* constitute a diverse group of secondary metabolites, including hyperoside (flavonols), chlorogenic acid and protocatechuic acid (hydroxycinnamic acids), procyanidins (flavan-3-ol oligomers), epicatechin (flavan-3-ols), isoquercitrin, vitexin, flavones, and flavonol glycosides. These compounds exhibit notable bioactive structures and properties, and the plant’s high phenolic acid content enhances food products [8,35].

In this research project, we extracted and investigated, for the first time, the phenolic compounds from a lyophilized butanolic fraction of *C. monogyna* comprising fruits, flowers, and leaves. We elucidated the total phenolic, total flavonoid, and flavonol content, determined the antioxidant and enzyme inhibitory properties, and characterized the chemical composition (phenolic and flavonoid substances) using LC-ESI-MS/MS. Various techniques, such as solid–liquid extraction and ultrasound-assisted extraction, were used to recover and isolate phenolic compounds from *Crataegus* species. Moreover, solvents of differing polarities, including methanol, ethanol, acetone, and n-butanol, were utilized. Despite limited previous studies on *Crataegus monogyna*, this research represents the first comprehensive examination of the photochemical compositions of its butanolic fraction derived from fruits, flowers, and leaves. The use of butanol as an extracting solvent for *C. monogyna* is novel. The reason for focusing on the butanolic fraction of *C. monogyna* is that this fraction has not been previously studied in the scientific literature. This provides a unique opportunity to explore new aspects, extract compounds with improved quantity and quality, and discover potential bioactive properties that have not yet been widely investigated.

**Figure 1:**
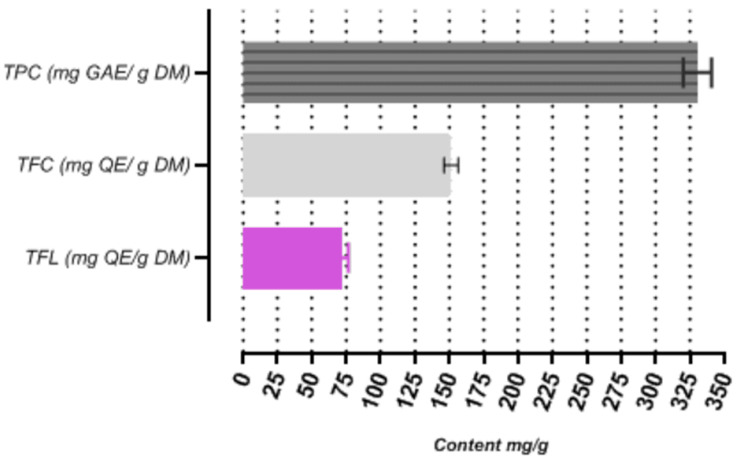
Total bioactive compounds of the BuOH fraction from *C. monogyna Jacq.* Bars represent standard deviation.

As observed in Figure 1, the lyophilized butanolic fraction yielded high concentrations of polyphenols, flavonoids, and flavonols. Accordingly, the total phenolic content (TPC) of the butanolic fraction obtained in this research project was measured to be 330 ± 0.69 mg GAE/g DW, the total flavonoid content (TFC) was found to be 151.38 ± 1.05 mg QE/g DW, and the total flavonol content (TFL) was determined to be 71.58 ± 00 mg QSE/g DW. The TPC was determined from a regression equation of the calibration curve and was expressed in gallic acid equivalents: y = 0.0034x + 0.1044 with R^2^ = 0.9972. The TFC was determined following the calculation of the calibration curve for quercetin: y = 0. 0048x, R^2^ = 0.997. The concentration of flavonols was determined similarly, using a calibration curve based on the quercetin standard. These values were then compared to those obtained from extracts of different *Crataegus* species from various regions worldwide, using different extraction solvents and analytical techniques.

The TPC of the *C. monogyna* butanolic fraction was higher compared to the methanolic extract obtained from the leaves with flowers (245.78 mg of GAE/g), fruit peel (93.43 mg of GAE/g), and seed (44.30 mg of GAE/g) of *C. monogyna* in Turkey [36]. Similar results were reported by another study of ethanolic extracts of *C. monogyna* from Gaziantep, Turkey, with TPC values for flowers (72.54 ± 2.58 mg GAE/g DM), leaves (86.88 ± 1.85 mg GAE/g DM), and fruits (71.69 ± 1.45 mg GAE/g DM) [37]. Moreover, the TPC and TFC in this study are significantly higher compared to those for methanol extracts from aerial parts of *C. monogyna* collected in Konya, Turkey, which reported a TPC of 68.13 ± 0.34 mg GAE/g and a TFC of 36.91 ± 0.17 mg QE/g [38]. Another recent study from Turkey reported TPC values for hydroalcoholic extracts of *C. monogyna* as follows: flowers (301.77 ± 21.8 mg GAE/g), leaves (169.66 ± 12.34 mg GAE/g), and fruits (116.24 ± 10.83 mg GAE/g) [39], which are approximately similar to our findings.

Additionally, methanol extracts from Romania’s *C. monogyna* showed lower values for polyphenols and flavonoids across different plant parts (flowers, leaves, ripened fruits, and immature fruits) with the TPC ranging from 105.10 to 284.06 µg EqGA/mg and the TFC from 5.12 to 41.28 μg QEs/mg extract [40]. Our results significantly exceed the TPC and TFC values reported for *C. monogyna* fruit methanol extracts from Iran (35.85 ± 0.25 mg GAE/g DW and 5.77 ± 0.09 mg QUE/g DW, respectively) [19]. A research team from Serbia noted lower values of TPC (14.9 ± 0.7 mg GAE/g) and TFC (3.51 ± 0.05 mg RUE/g) in ethanolic extracts of *C. monogyna* fruits [41]. Conversely, our values are in line with the previous results of [42], which showed that the n-butanol extract of the Algerian *C. azarolus* species (aerial parts) had a high total phenolic and flavonoid content, with values of 307.33 ± 2.33 mg GAE/g extract and 143.0 ± 2.12 mg QE/g extract, respectively. Similarly, high amounts of total polyphenol, total flavonoid, and flavonol content in the Algerian *C. oxyacantha* fruit and leaf butanolic extracts are as follows: in fruits (136.54 ± 8.52 µg GAE/mg extract, 21.46 ± 0.5819 µg QE/mg extract, and 21 ± 0.29 µg QE/mg extract) and in leaves (521.05 ± 2.70 µg GAE/mg extract, 116.176 ± 0.59 µg QE/mg extract, and 45.96 ± 1.99 µg QE/mg extract), respectively [43]. A study from Spain reported lower polyphenol content (78 ± 4 mg GAE/g DW) in *C. monogyna* leaves obtained by UAE extraction using acetone/water [6]. According to the Pearson’s correlation coefficient, the total flavonol content (TFL) was strongly correlated with the TPC (r = 0.9922) and TFC (r = 0.9342). Moreover, a strong correlation was found between TPC and TFC (r = 0.9585). These correlations are shown in Table 1.

Several factors influence the content of phenolic compounds in *Crataegus* species, including intrinsic factors such as species and variety, plant part, and maturation stage [44], and extrinsic factors such as climatic, environmental, and ecological conditions [45]. Furthermore, differences in solvents and techniques used for extractions can also be significant factors that specifically influence the qualitative and quantitative composition of phenolic compounds in *Crataegus* species extracts [46].

Although the proposed extraction method has proven effective at the laboratory scale, the issue of scalability has not been addressed. This discussion is crucial as it helps assess whether the method can be applied on a larger industrial scale. Potential challenges in scaling up may include issues with material availability, production costs, and quality assurance. Therefore, discussing these challenges and proposing appropriate solutions can enhance our understanding of the method’s applicability on a larger scale.

### 3.2. LC-ESI-MS/MS Evaluation

To confirm the aforementioned results and gain a deeper understanding of the nature of the bioactive compounds, we identified and characterized the phytochemicals molecules present in the lyophilized *C. monogyna* butanolic extract.

The developed LC-ESI-MS/MS technique used in this research was performed following the validated method established by Yilmaz (2020). This technique is noted for its superior selectivity and sensitivity, making it more distinctive than other liquid chromatography methods [3,22].

**Figure 2:**
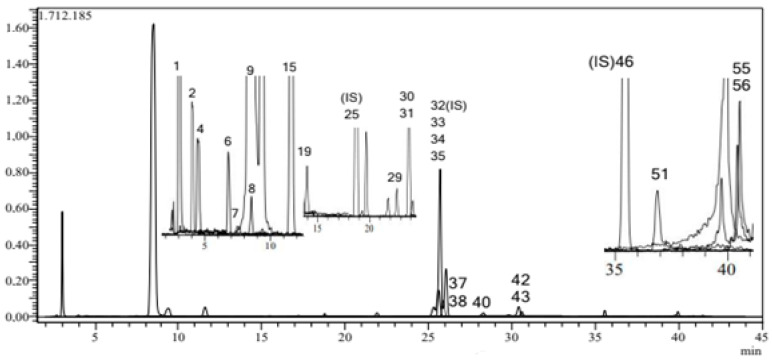
LC-ESI-MS/MS chromatogram of butanolic fraction from *C. monogyna.* 1: quinic acid; 2: fumaric acid; 4: gallic acid; 6: protocatechuic acid; 7: catechin; 8: gentisic acid; 9: chlorogenic acid; 15: epicatechin; 19: vanillin; 25: ferulic acid-D3-IS; 29: salicylic acid; 30: cynaroside; 31: miquelianin; 32: rutin-D3-IS; 33: rutin; 34: isoquercitrin; 35: hesperidin; 37: genistin; 38: rosmarinic acid; 40: cosmosiin; 42: astragalin; 43: nicotiflorin; 46: quercetin-D3-IS; 51: genistein; 55: chrysin; and 56: acacetin. The chromatogram of the standards is shown in Figure S1 (Supplementary Material).

Overall, 23 fingerprint phytochemical compounds were identified and quantified in the *C. monogyna* butanolic extract: eight flavonoid glycosides (flavanone), eight phenolic acids, two flavonoid aglycones (flavone), two flavan-3-ols (flavanols), two isoflavon glycosides, and one phenolic aldehyde. The principal peak chromatogram (PPC) of *the C. monogyna* extract is shown in Figure 2, while the quantitative results are provided in Table 2. Analytical method validation parameters for the LC-ESI-MS/MS method are summarized in Table S1 (Supplementary Material).

**Figure 3:**
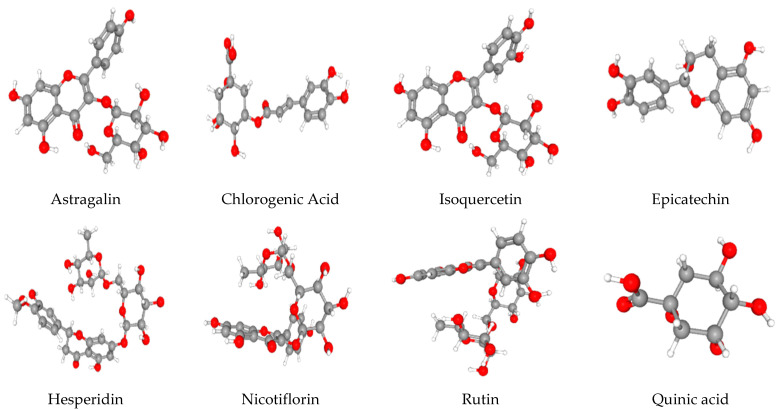
3D structures of the higher quantified phenolic molecules in *C. monogyna* butanolic extract obtained from the PubChem database.

It was evident that the highest quantified phenolic molecules were: epicatechin (flavan-3-ols) at 99.916 ± 2.208 mg/g, isoquercitrin at 53.31 ± 1.172 mg/g, chlorogenic acid at 47.457 ± 1.010 mg/g, quinic acid at 37.819 ± 1.406 mg/g, rutin at 29.98 ± 0.740 mg/g, hesperidin at 5.296 ± 0.177 mg/g, astragalin at 1.774 ± 0.020 mg/g, and nicotiflorin at 1.482 ± 0.016 mg/g. Their 3D structures are presented in Figure 3.

Regarding the constituents of phenolic compounds, the butanolic extract of *C. monogyna* represents a substantial source of flavanols, phenolic acids, and glycosidic flavonoids.

#### 3.2.1. Epicatechin

Epicatechin (as shown in Figure 3), a significant polyphenolic compound from the flavan-3-ols subclass [47], is identified as being among the predominant constituents in C. *monogyna*. It is a key bioactive compound found in various plant-derived foods such as green tea leaves, cocoa, apples, berries, grapes [48], nuts, and seeds, and in processed products like red wine and dark chocolate [49]. For the first time, this research project has found that the butanolic extract of *C. monogyna* serves as a significant reservoir of epicatechins. When compared to recent and older studies on *C. monogyna* species, this study demonstrates higher concentrations. Therefore, as reported by [50], a low concentration of (-)-Epicatechin was quantified in phenolic extracts from *C. monogyna* buds and fruits, measuring 2.32 ± 0.08 mg/g dw and 5.19 ± 0.59 mg/g dw, respectively, using HPLC-DAD-ESI/MS methods. In contrast, a study conducted by [6] reported that the concentration of monomeric catechin/epicatechin in *C. monogyna* leaves, obtained by UAE extraction using acetone/water and quantified by HPLC-ESI-TOFMS and HPLC-FLD analysis, was 8.7 ± 0.2 mg/g dw. This result is in agreement with the findings of Herrera et al. from Spain, where the epicatechin content in wild fruits from *C. monogyna* aqueous extract, using HPLC-MS analysis, measured 8.12 ± 1.03 mg/g extract [51], which is notably lower than the concentrations obtained in our study.

In addition, researchers from Romania [52] determined the epicatechin content in *C. monogyna* fruit ethanolic extract to be 143.25 ± 10.64 mg/100 g DW using HPLC-UV analysis. In a study by [39] on *C. monogyna* species, epicatechin concentrations were estimated using HPLC, with a total of 36.75 mg/g found in the hydroalcoholic extract, including 17.56 ± 0.27 mg/g in flower-bearing branches, 5.95 ± 0.11 mg/g in leaves, and 13.24 ± 0.18 mg/g in fruits. Current investigation has revealed an unprecedentedly high concentration of epicatechin in the Algerian *C. monogyna* species. Using the LCMS/MS system, we measured a content of 99.916 ± 2.208 mg/g DW, marking the highest concentration of this compound recorded in this species to date. This surpasses the levels found in many prominent plants known for their richness in epicatechin, such as cacao and broad bean pods, which have mean concentrations of 70.36 mg/100 FW and 37.55 mg/100 FW, respectively, according to recent studies by Prakash et al. (2019) [53].

These results confirm that the Algerian *C. monogyna* species could serve as a significant natural source of epicatechin, a compound demonstrating diverse pharmacological activities such as cardioprotective, antidiabetic, and neuroprotective [48], enhancing the structure and function of mitochondria [54]. Epicatechin also possesses anticancer, antiviral, and anti-inflammatory properties [55]. Additionally, it is utilized in the cosmetics industry as a skincare product [56] and is considered a functional antioxidant in the food industry, specifically as a food additive [49].

#### 3.2.2. Isoquercitrin

As presented in Figure 3, isoquercitrin was identified as the substance with the second-highest level in the extract, at 53.31 ± 1.172 mg/g DW. Isoquercetin is a natural flavonoid compound and the 3-O-β-D-glucoside form of quercetin, with a glucose unit attached, and it is widely found in plants such as buckwheat (*Fagopyrum esculentum*), green tea (*Camellia sinensis*), St. John’s Wort (*Hypericum perforatum*), onions (*Allium cepa*), ginger (*Zingiber officinale*), and grapes, though its concentration levels can vary [57]. Moreover, in hawthorn, isoquercitrin is one of several glycosides and phenolic compounds that contribute to its health benefits. Additionally, the *C. monogyna* species is known to contain isoquercitrin, along with other flavonoids [35]. Furthermore, compared to previous studies that have focused on the identification and quantification of isoquercetin in *Crataegus* species using various chromatographic techniques and solvent extracts, our research project is the first to study the butanolic extract of *C. monogyna* to identify and quantify the isoquercitrin compound, where the highest concentration recorded was 53 mg/g DW. Since this extract had not been previously studied in this species of *Crataegus*, we were compelled to compare our findings with studies on the same species but using different extract fractions. Accordingly, this value is higher than those obtained from the ethanolic extract of *C. monogyna Jacq* fruit obtained by ultrasound extraction using HPLC–MS-ESI-TOF analysis, which reported 188.61 ± 26.96 μg/g DW of quercetin 3-O-glucoside isomer b (isoquercitrin) [58]. Furthermore, a low isoquercitrin (quercetin 3-β-D-glucoside) content of 40.08 ± 3.10 μg/g DW was quantified in the ethanolic extract of fresh ripe fruits of *C. monogyna* by accelerated solvent extraction using HPLC-DAD analysis, as reported by Paun and collaborators [59]. On the other hand, the only high content of isoquercitrin found in previous studies was reported by Turnalar Ülger and his team, with a value of 9.28 mg/g DW in the flower-bearing branches, leaves, and fruits from *C. monogyna* hydroalcoholic extract using HPLC analysis [39]. However, these values are significantly lower compared to what we have found. Accordingly, our results confirm that Algerian *C. monogyna* species could serve as a significant natural source of isoquercitrin.

Among the most extensively studied types of flavonoids, isoquercitrin has numerous beneficial properties, including antioxidant, anti-obesity, and anti-inflammatory effects, which are beneficial in preventing chronic diseases such as cardiovascular diseases, diabetes, and cancer. Studies have shown that isoquercitrin can reduce oxidative stress and inflammation in cells and its glucose moiety enhances the absorption of quercetin in the body, increasing its bioavailability. Additionally, isoquercitrin has shown promising potential in improving cognitive functions and reducing the growth of cancer cells in laboratory and animal studies. As an antiviral agent, isoquercitrin (Q3G) is also a candidate for in vitro investigation against COVID-19 [60]. Generally, it is considered as an ingredient in dietary supplements and nutraceuticals due to its beneficial health properties. These properties make it useful in pharmaceutical and nutritional applications to support overall health and prevent disease [8,61].

#### 3.2.3. Chlorogenic Acid

As shown in Figure 3, chlorogenic acid was identified as the substance with the third highest level in the butanolic extract, at 47.457 ± 1.010 mg/g DW. This polyphenolic compound, synthesized via the shikimic acid pathway during aerobic respiration, is formed through the esterification of caffeic acid and quinic acid. It is widely distributed among higher dicotyledonous plants, ferns, and numerous traditional Chinese medicinal plants, including green coffee beans, which contain about 6 to 12% *w*/*w* total chlorogenic acids (CGAs) [62]. Besides coffee, *Crataegus* species stand out for hosting high concentrations of chlorogenic acid, ranging from 11.9 to 54.4 mg/100 g on a wet basis [63].

For the first time, this research project has demonstrated that the *C. monogyna* butanolic extract contains the highest amount of chlorogenic acid compared to recent studies. This value surpasses those obtained from the fruit, leaves, and flowers of *C. monogyna* species, which reported 41.75 ± 3.78 mg/100 g DW, 508.3 ± 24.74 mg/100 g DW, and 226.3 ± 18.31 mg/100 g DW, respectively [52]. Furthermore, a low chlorogenic acid content of 6.51 ± 0.09 mg/g DW was quantified in the *C. monogyna* leaf extract using HPLC-ESI-TOFMS and HPLC-FLD, as reported by Martín-García and colleagues [6]. In contrast, Turnalar Ülger and his collaborators found a high chlorogenic acid content of 45.52 ± 0.50 mg/g in flower-bearing branches and 40.89 ± 0.44 mg/g in leaves from the *C. monogyna* hydroalcoholic extract using HPLC analysis, which are approximately consistent with our findings [39].

Chlorogenic acid is widely recognized as one of the most prevalent phenolic acid compounds in foods. It is esteemed as a nutraceutical due to its potential health benefits against chronic metabolic diseases, including cardiovascular diseases, diabetes mellitus, and neurodegenerative disorders, as well as its anti-inflammatory effects. Additionally, it serves as a natural food additive in the food industry due to its antimicrobial properties against a broad spectrum of microorganisms, including, bacteria, yeasts, molds, viruses, and amoebas. Its antioxidant effects also help inhibit lipid oxidation, thereby extending the shelf life of food products [62].

#### 3.2.4. Quinic Acid

Quinic acid, identified as the fourth most abundant phenolic compound in the butanolic fraction of *Crataegus monogyna*, was found at a concentration of 37.819 ± 1.406 mg/g DW (see Figure 3). It is derived from the hydrolysis of chlorogenic acid [64], with a strong correlation being observed between these two compounds [65]. Quinic acid is present in various plants [66], and has notable therapeutic properties, including antidiabetic, antiviral, anticancer, and anti-inflammatory effects [65]. Additionally, it is used in food production for its antioxidant and antibacterial properties, aiding in product shelf life extension [67]. Our study highlights that the butanolic extract of *C. monogyna* contains significant levels of quinic acid, ranking as the second most abundant organic acid in the extract, after chlorogenic acid. This high concentration is attributed to its role in plant biosynthetic processes, serving as a precursor for producing polyphenols like chlorogenic acids and flavonoids [68]. To date, research on quinic acid in Crataegus species, especially *C. monogyna*, has been limited. Existing studies primarily focus on identifying quinic acid and its derivatives using various extraction methods and analytical techniques. Notably, [69] characterized quinic acid and its derivatives from *C. monogyna* leaves and fruits using UPLC-ESI-Q-TOF-MS/MS. Similarly, research from Portugal identified quinic acid and its derivatives in *C. monogyna* aerial parts using HPLC-PDA-ESI/MS [4].

Recent studies by Razola-Díaz and collaborators from Poland identified derivatives of quinic acid, such as coumaroylquinic and dicaffeoylquinic acids, in *C. monogyna* leaf and flower extracts using HPLC–MS ESI-TOF techniques [58]. Our research project is the first to characterize and quantify quinic acid in the butanolic extract of *C. monogyna* using LC-MS/MS techniques. Compared to the existing literature, our study reports the highest concentration of quinic acid. Rodrigues et al. found lower concentrations of quinic acid derivatives, such as 3-O-caffeoylquinic acid and 4-O-caffeoylquinic acid, at 7.16 mg/g and 2.57 mg/g, respectively, in *C. monogyna* flower buds and fruit [50]. Similarly, Mraihi et al. reported lower concentrations of quinic acid derivatives in *C. monogyna* fruit [70]. The levels reported in our findings are also higher than those reported by Herrera et al., who quantified quinic acid derivatives in wild fruits of *C. monogyna* at 1.19 mg/g extract [51].

#### 3.2.5. Rutin

Rutin, the fifth most abundant compound in the butanolic extract of *C. monogyna*, was identified at a concentration of 29.98 ± 0.740 mg/g DW (see Figure 3). Also known as quercetin-3-O-rutinoside, rutin is a significant plant-derived flavonoid with antioxidant and anti-inflammatory properties. It supports cardiovascular health by strengthening capillaries and improving their elasticity. Additionally, rutin has anticancer and antidiabetic effects and is widely used in dietary supplements, pharmaceuticals, and cosmetics for its protective effects on cells and skin health [71]. In *Crataegus* species, rutin is a significant flavonoid that enhances the therapeutic value of *Crataegus* in both traditional and modern medicine, making it beneficial for managing heart health and preventing oxidative damage [35]. Our research focused on the butanolic fraction of *C. monogyna,* which demonstrated the highest amount of rutin compared to recent studies on different *Crataegus* species extracts from various plant parts (flowers, leaves, and fruits) using various analytical techniques. Notably, Turnalar Ülger and his team detected and quantified rutin with a concentration of 2.01 mg/g in the flower-bearing branches and leaves of *C. monogyna* from a hydroalcoholic extract using HPLC analysis. Rutin was not detected in the fruits in this study [39]. In contrast, fresh ripe fruits of *C. monogyna*, extracted with ethanol using accelerated solvent extraction and analyzed by HPLC-DAD, revealed a significant rutin content of 2.927 mg/g [59]. Both of these values are lower than those in our findings. Accordingly, based on this research, we can conclude that *C. monogyna* is a significant reservoir of rutin.

#### 3.2.6. Hesperidin

Hesperidin, the sixth most concentrated compound identified in the *C. monogyna* butanolic extract, was found at 5.296 ± 0.177 mg/g DW (see Figure 3). Hesperidin is a bioflavonoid belonging to the flavanone category, and is also known as a type of vitamin P. It is abundantly found in citrus peel fruits like lemons and sweet oranges (*Citrus sinensis*)*,* contributing to their bitter flavor, and to a lesser extent in grapefruits, tomatoes, mint, and other fresh herbs [72]. To the best of our knowledge, hesperidin has not yet been detected or quantified in *Crataegus* species. In this research project, an unexpected concentration of hesperidin was revealed from the butanolic extract of Algerian *C. monogyna* using the LC-ESI-MS/MS technique, known for its high accuracy and ability to detect compounds in very precise quantities. This discovery is notable because hesperidin is typically associated with citrus fruits and is not commonly found in significant amounts in *Crataegus* species.

Previous research on *Crataegus* species has primarily focused on flavonoids and other phenolic compounds, including oligomeric proanthocyanidins, organic acids, triterpene acids, vitamin C, and other bioactive molecules. The discovery of hesperidin adds to this list of bioactive compounds, potentially explaining some of the significant health benefits observed in the traditional uses of hawthorn that were not fully understood. Hesperidin is known to exhibit a wide range of pharmacological activities, including antioxidant and anti-inflammatory actions, and protective effects against cardiovascular disease, diabetes, and cancer. Recently, hesperidin has also been investigated for its potential antiviral properties, specifically against coronavirus disease (COVID-19) [73,74]. Due to its many functional activities, hesperidin finds extensive use in the food and pharmaceutical industries due to its natural antioxidant properties, its antimicrobial activities, and as a biofunctional additive to extend product shelf life and improve the bioavailability of pharmaceutical drug formulations [75]. The presence of a significant concentration of hesperidin in Algerian *C. monogyna* suggests previously unrecognized phytochemical diversity in this species. This diversity could arise from a complex interplay of environmental, genetic, chemical, and analytical factors, leading to the synthesis of new and beneficial compounds. These findings enhance our understanding of the potential health benefits associated with *C. monogyna* species and could expand their applications in medicine, functional foods, and the food industry as biofunctional additives, as well as in pharmaceutical products, including the development of novel drugs.

#### 3.2.7. Astragalin

Astragalin, identified as the seventh most abundant compound in the butanolic extract of *C. monogyna*, was quantified at 1.774 ± 0.020 mg/g DW, as shown in Figure 3. It is a natural flavonoid formed by the glycosylation of kaempferol. Astragalin is found in several plants, including *Astragalus membranaceus*, white mulberry, green tea seeds, and species of the Rosaceae family [76]. *Crataegus* species, such as *C. monogyna,* indicate a relatively high amount of astragalin, suggesting it as a rich source of this bioactive compound compared to findings from other research projects. Mraihi et al. detected and quantified Kaempferol-3-O-glucoside (astragalin) measuring 1.491 mg/g DW in the dried peel of *C. monogyna* red fruit extract using HPLC–MS analysis. Astragalin was not detected in the pulp and seed [70], which is approximately in accordance with the results of our study. Additionally, our content of astragalin was much higher compared to that obtained from *C. monogyna* leaf extract by HPLC-MS using ultrasonic-assisted extraction, which had a value of 0.0471 ± 0.0002 mg/g DW [6]. Similarly, a low concentration of astragalin (kaempferol-3-O-glucoside) was quantified in the phenolic ethanolic extract from *C. monogyna* fruit (measuring 0.04258 ± 0.68 mg/g DW) using UHPLC/(-)HESI–MS/MS methods [41]. Recent research by [58] also determined a low concentration of astragalin in *C. monogyna* fruit ethanolic extract (0.01107 mg/g DW) using ultrasound extraction and the HPLC–MS-ESI-TOF analysis method, which further reinforces our findings. Furthermore, some studies have merely identified the presence of astragalin in *Crataegus* species. For instance, Wang et al. detected kaempferol-3-O-glucoside (astragalin) in hawthorn fruits’ methanol extract using ESI-Q TRAP-MS/MS [77]. Conversely, others have not found this compound in the polyphenolic composition of this species. For example, [78] analyzed *C. monogyna* flowers’ methanolic extract using HPLC-DAD-ESI/MS analyses, and [13] examined ripe fruits and aerial parts (leaves and stems) of *C. monogyna* from Chile using LC-DAD, LC–MS, and MS/MS. The significant presence of astragalin in *C. monogyna* makes this species a valuable resource for the development of natural treatments and innovative health products. The exceptional biological activities of astragalin and its potential therapeutic effects include anti-inflammatory, antioxidant, and antibacterial properties [76], covering a wide range of diseases such as cancers, obesity, diabetes and its complications, respiratory diseases, neurological disorders such as AD, and reproductive system diseases [79].

#### 3.2.8. Nicotiflorin

Nicotiflorin, a significant phenolic compound in the butanolic extract of *C. monogyna*, shown in Figure 3, was quantified at 1.482 ± 0.016 mg/g DW. This flavonoid glycoside (kaempferol-3-O-rutinoside) is naturally found in plants and is derived from *Carthamus tinctorius* (safflower) in traditional Chinese medicine. It is chemically related to astragalin as a glycoside of kaempferol [80]. To our current knowledge, this is the first research project where such a significant concentration of nicotiflorin has been obtained from the *Crataegus* species, particularly *C. monogyna.* The only study we found, conducted by Barros et al., detected and quantified nicotiflorin in *C. monogyna* at a value of 0.64 ± 0.03 mg/g using HPLC-DAD-ESI/MS technique [78]. This significant content of nicotiflorin in *C. monogyna* offers numerous health benefits due to its antioxidant, anti-inflammatory, liver-protective, and brain-protective properties. Its applications span pharmaceuticals, nutraceuticals, cosmetics, and traditional medicine [80]. Furthermore, our study revealed that the *C. monogyna* butanolic extract contains a range of components in moderate concentration, including cosmosiin (0.994 ± 0.008 mg/g), cynaroside (0.758 ± 0.027 mg/g), fumaric acid (0.540 ± 0.004 mg/g), gallic acid (0.249 ± 0.002 mg/g), and catechin (0.308 ± 0.006 mg/g). These compounds are less concentrated but still contribute to the overall profile of the extract. For example, gallic acid is known for its antioxidant and antimicrobial properties [81]. Catechin, while at a moderate level, is recognized for its cardiovascular benefits and antioxidant effects [82], as shown in Table 2. Additionally, low concentrations (trace) of the compounds chrysin, acacetin, gentisic acid, protocatechuic acid, salicylic acid, miquelianin, rosmarinic acid, and genistin were detected in the butanolic extract studied, with content levels ranging between 0.010 ± 0.0001 and 0.069 ± 0.001 mg/g DW, as shown in Table 2. These compounds may still offer valuable insights into the overall chemical profile of the extract, though their lower levels might limit their impact compared to the higher concentration compounds. Furthermore, aconitic acid, epigallocatechin, protocatechuic aldehyde, tannic acid, epigallocatechin gallate, cynarin, 4-OH benzoic acid, vanilic acid, caffeic acid, syringic acid, syringic aldehyde, daidzin, epicatechin gallate, piceid, p-coumaric acid, ferulic acid, sinapic acid, coumarin, o-coumaric acid, ellagic acid, quercitrin, fisetin, daidzein, quercetin, naringenin, hesperetin, luteolin, kaempferol, apigenin, and amentoflavone were not detected in the butanolic extract, as shown in Table 2. Furthermore, the study reveals significant findings regarding the elevated levels of epicatechin, isoquercitrin, chlorogenic acid, quinic acid, rutin, hesperidin, astragalin, and nicotiflorin in Algerian *Crataegus monogyna*. These results indicate that this plant serves as a valuable natural source of these important biomolecules. Additionally, the study highlights the importance and effectiveness of the extraction methods used, emphasizing the richness of the butanolic extract in phenolic compounds compared to other fractions reported in the literature. The study also underscores the comprehensive and validated LC-ESI-MS/MS analytical techniques employed for detecting and quantifying phenolic concentrations.

### 3.3. Antioxidant Properties

The abundance and diversity of bioactive compounds in *C. monogyna* significantly enhance its antioxidant properties, making it an effective natural antioxidant source. These compounds work synergistically to neutralize free radicals and absorb oxygen radicals, thereby boosting the total antioxidant potential of the plant. The presence of various antioxidants provides a more comprehensive defense against oxidative stress as different compounds may target multiple pathways and mechanisms of free radical formation and propagation [83]. This aspect has garnered increasing interest among researchers due to its potential applications in food, cosmetics, and health sciences.

In this study, the antioxidant properties of the lyophilized butanolic fraction of *C. monogyna* were evaluated using five different antioxidant tests: DPPH, GOR, and ABTS assays to assess radical scavenging activity, along with CUPRAC and reducing power assays, which involve various reaction mechanisms and conditions.

These assays, including DPPH and ABTS, directly measure the ability of antioxidants to donate hydrogen atoms or electrons in controlled, lipid-free systems. They offer valuable insights into the intrinsic antioxidant potential of substances, minimizing interference from environmental factors. In contrast, the GOR test stands out by employing gas-phase radicals, simulating more complex environmental conditions compared to the solution-based DPPH and ABTS assays. This method is particularly useful for assessing antioxidants in complex matrices such as foods or biological samples, where oxidation processes can occur in the gas phase rather than in simple solution-based systems. Additionally, the redox potential or reducing power of antioxidants serves as a critical indicator of their antioxidant effectiveness, evaluated through redox reactions involving various metal ions such as iron, copper, chromium, and cerium. The CUPRAC and reducing power tests are commonly employed in this context [84].

Our in vitro research demonstrated substantial antioxidant activity across all tests, benchmarked against various standards, including Trolox, BHT, BHA, ascorbic acid, and *α*-tocopherol. Specifically, the results were as follows: GOR (IC50 = 3.82 ± 0.29 µg/mL), ABTS (IC50 = 4.98 ± 0.20 µg/mL), CUPRAC (A0.5 = 10.93 ± 0.16 µg/mL), reducing power (A0.5 = 14.78 ± 0.86 µg/mL), and DPPH (IC50 = 16.80 ± 0.64 µg/mL). These results range from the highest activity observed in the GOR assay to the lowest in the DPPH assay.

To the best of our knowledge, this study represents the first investigation into the antioxidant activities of the butanolic fraction of *C. monogyna*. There are no published reports in the literature specifically studying this butanolic fraction and these specific antioxidant tests, as detailed in Table 3. The terms “IC50” and “A0.50 levels” denote the concentration needed to attain 50% inhibition and the concentration at which the absorbance reaches 0.50, respectively. These values were determined using a linear regression approach and are expressed as mean ± SD *(n* = 3).

Given the novelty of our research, we compared our findings with the existing literature that focuses on various extracts of the same plant (*C. monogyna*). Notably, Maammeri et al. (2022) reported that the methanolic extract of *C. monogyna* leaves exhibited weaker activities with DPPH (28.00 ± 0.97 μg/mL), ABTS (10.12 ± 0.60 μg/mL), GOR (36.84 ± 1.85 μg/mL), and CUPRAC (28.79 ± 12.10 μg/mL) assays, in contrast to our findings [85]. Furthermore, our test showed that the butanolic extract of *C. monogyna* (leaves, flowers, and fruits) recorded higher radical scavenging activity compared to recent research by Özderin (2024) on *C. monogyna var. odemisii* fruit ethanol extract, which had DPPH IC50 18.31 ± 0.45 μg/mL, ABTS IC50 9.25 ± 0.36 μg/mL, and CUPRAC IC50 15.74 ± 0.55 μg/mL [84]. On the other hand, Keser et al. investigated the antioxidant capacities of ethanol extracts of *C. monogyna subsp. monogyna Jacq* (hawthorn) from flowers, leaves, and fruits. They conducted tests using DPPH, ABTS, and reducing power assays, with BHA, BHT, tocopherol, and Trolox as standards. The results showed DPPH values of 58.15 ± 0.45, 67.57 ± 0.89, and 33.24 ± 0.28 at 100 μg/mL concentration for flowers, leaves, and fruits, respectively. For ABTS, values were 97.90 ± 0.71 and 97.60 ± 0.46 for flowers and leaves, and 52.50 ± 0.56 for fruits, all at 100 μg/mL concentrations. The reducing power values were 0.102, 0.076, and 0.109 at 100 μg/mL concentration for flowers, leaves, and fruits, respectively. These findings indicate weaker antioxidant activity compared to our results [37].

Notably, the butanolic extract of *C. monogyna* possesses highly effective antioxidant properties, which are attributed to its richness in high concentrations of polyphenolic active compounds. These compounds efficiently counteract free radicals and shield cells from oxidative damage associated with various diseases. This is supported by the Pearson’s correlation coefficient shown in Table 1. We observed a strong correlation between the content of total phenolic, total flavonoid, and total flavonol compounds, and antioxidant activities (DPPH and ABTS). There was a highly significant positive correlation between TPC and DPPH activity (r = 0.9580), and a significant positive correlation between TPC and ABTS activity (r = 0.5214). Moreover, a very strong correlation was found between TFC and DPPH activity (r = 1), and TFC correlated strongly with ABTS activity (r = 0.7201). Similarly, there was a very strong correlation between TFL and DPPH activity (r = 0.9922), as well as ABTS activity (r = 0.7517). This confirms and strengthens the hypothesis that the quantity and types of polyphenols (epicatechin, chlorogenic acid, quinic acid, hesperidin, astragalin, nicotiflorin, and others fingerprinted phytochemicals compounds) in a *C. monogyna* butanolic extract are critical determinants of its antioxidant activity.

Therefore, due to its richness in several compounds at very high concentrations, especially those known for their food preservation properties such as epicatechin, chlorogenic acid, quinic acid, hesperidin, and astragalin, and its highly potent antioxidant properties, these findings lead us to classify the Algerian *C. monogyna* plant as rich in natural antioxidants. This opens avenues for its application not only in medicine but also in the pharmaceutical, cosmetic, and food sectors, particularly as a natural antioxidant alternative to synthetic antioxidants, including BHA and BHT. Our results demonstrate that the butanolic extract of *C. monogyna* is more effective than BHA, thus, potentially mitigating their side effects, potential health risks, and toxicity, given its demonstrated high effectiveness to extend product shelf life [8].

### 3.4. Enzymatic Inhibitory

#### 3.4.1. ACHE Inhibitory

Plants remain a crucial source in the discovery and development of new treatments for various diseases that have long burdened patients and challenged researchers in the medical field. They offer promising alternatives to synthetic drugs, which often carry numerous side effects, are costly, pose various health difficulties, and have limited accessibility. Continuous research is crucial to fully exploit these natural resources’ therapeutic potential and overcome the challenges associated with their use. Among the most significant of these diseases is Alzheimer’s disease, which is considered the most common neurodegenerative disease worldwide. The primary pharmacological strategy currently employed to treat Alzheimer’s disease involves inhibiting the acetylcholinesterase (AChE) enzyme. This approach plays a crucial role in symptomatic treatment by preventing the breakdown of acetylcholine, thereby addressing neurotransmitter imbalances [86].

The phenolic compounds of *C. monogyna,* due to their variety and potential antioxidant properties, help fight against free radicals and reduce brain cell damage. Evaluating their potential to stabilize AChE function (in vitro) would yield valuable findings. Accordingly, the anticholinergic activity of *C. monogyna* butanolic extract was examined for its ability to inhibit acetylcholinesterase (AChE). The results are presented as IC50 (µg/mL) (Table 4).

As illustrated in Table 4, the AChE inhibitory activity of *C. monogyna* butanolic extract was determined and compared to that of galantamine (used as standard), with an IC50 value of 34.75 ± 1.99 µg/mL. The IC50 value for AChE inhibition by *C. monogyna* butanolic extract was determined to be 43.65 ± 2.10 µg/mL. Statistical analysis revealed that these values differed significantly (*p* < 0.05), indicating a noteworthy disparity in the inhibitory efficacy between the plant extract and the standard compound. Although the butanolic extract is less effective per unit compared to galantamine, its requirement for a smaller quantity of the natural product could offset this difference. Consequently, when considering the amount used, the butanolic extract could be as effective as, or potentially more effective than, galantamine.

These findings can be attributed to bioactive compounds in the extracts that effectively inhibit the activity of AChE. Additionally, this has been confirmed by the Pearson’s correlation coefficient shown in Table 1. We observed a strong correlation between the content of total phenolic, total flavonoid, and total flavonol compounds, and AChE inhibitory activity. We found a highly significant positive correlation between TPC and AChE inhibitory activity (r = 0.9701). Moreover, a very strong correlation was determined between TFC and AChE inhibitory activity (r = 0.9989), and a very strong correlation was found between TFL and AChE inhibitory activity (r = 0.9885). These results confirm our findings, as the *C. monogyna* butanolic extract contains very high concentrations of epicatechin, isoquercetrin, rutin, and chlorogenic acid, among the most important compounds that have demonstrated effectiveness in AChE inhibition [62,87,88,89]. Furthermore, recent research has demonstrated that extracts from the *Crataegus* genus may exhibit the ability to inhibit acetylcholinesterase enzyme activity [35,90,91,92]. Particularly in the *C. monogyna* species, according to a previous study by [38], the aerial parts of *C. monogyna* methanol extract exhibited anticholinesterase activity with an IC50 > 200 μg/mL, which does not agree with our results. Another study evaluated the ability of a hydro-alcoholic solution of *C. monogyna* fruit to inhibit AChE enzyme with an IC50 value ranging between 10 µg/mL and 50 µg/Ml [93]. Notably, this high concentration of these bioactive compounds (epicatechin, isoquercetrin, rutin, and chlorogenic acid) not only underscores the potential of hawthorn as a natural source of AChE inhibitors but also suggests that it could be a valuable addition to medicinal and pharmaceutical applications. This provides a natural alternative to synthetic inhibitors for managing conditions like Alzheimer’s disease.

#### 3.4.2. Alpha-Amylase Inhibitory

**Figure 4:**
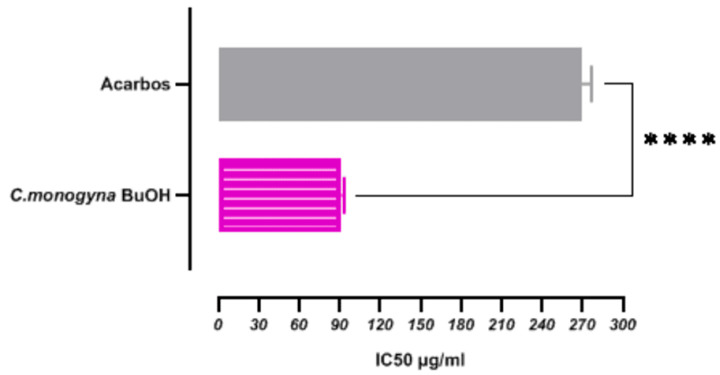
Alpha-amylase inhibitory activity of butanolic fraction from *C. monogyna Jacq.* and **** indicate that significant at *p* value < 0.0001.

Among the most significant chronic diseases of our time, affecting millions of people, is diabetes. This disease is characterized by elevated blood sugar levels due to problems with the production or effectiveness of insulin, which regulates blood sugar concentration. Enzymes are essential for the digestive process, and among these enzymes is alpha-amylase, which helps break down starches into simple sugars. This enzyme is mainly produced in the pancreas and salivary glands. Alpha-amylase inhibition is an essential topic in diabetes treatment studies to help control blood sugar levels [94]. Notably, synthetic inhibitors such as acarbose are clinically used to control hyperglycemia in type II diabetic patients. However, these drugs can cause adverse side effects, including irritable bowel syndrome, severe kidney or liver impairment, and drug resistance development. This has led to a search for natural and safer alternatives [95]. Natural compounds extracted from plants have shown promise as *α*-amylase inhibitors without having the side effects of synthetic drugs. In this context, we investigated the antidiabetic effect of *C. monogyna* butanolic extract for the first time, focusing on its inhibition of *α*-amylase both in vitro and in comparison to the standard drug acarbose. The findings are presented in Figure 4.

The IC50 value for *α*-amylase inhibition by *C. monogyna* butanolic extract was determined to be 91.19 ± 0.10 µg/mL, indicating its greater effectiveness compared to acarbose, which exhibited an IC50 value of 270.75 ± 1.99 µg/mL. Statistical analysis revealed that these values differed significantly (*p* < 0.05), indicating a noteworthy disparity in inhibitory efficacy between the plant extract and the standard reference compound. Hawthorn has a well-documented history of use in diabetes treatment. Numerous previous studies by [94,96,97,98,99,100,101,102,103,104] have investigated the efficacy of *Crataegus* species in treating diabetes complications through both in vivo and in vitro experiments. In vivo research projects using diabetic animal models have demonstrated significant improvements in blood glucose levels, reduced inflammation, and mitigation of various diabetes-related complications. Specifically, research on the *C. monogyna* species has examined its antidiabetic activity and the effects of *C. monogyna* extract in inhibiting the *α*-amylase enzyme. Notably, a previous study [38] reported that the methanolic extract of *C. monogyna* exhibited a lower inhibitory effect on *α*-amylase, with an IC50 greater than 500 μg/mL, compared to our findings. Furthermore, a study conducted in the Syrian Arab Republic by [105] on a methanolic extract of *C. monogyna* leaves reported an IC50 value of 125 µg/mL in autumn and an IC50 value of 148 µg/mL in spring, both of which are lower than our results. The antidiabetic activity of the butanolic extract of *C. monogyna* obtained in this study could potentially be attributed to its high content of phenolic compounds, flavonoids, and flavonols. This study identified a positive correlation between the antidiabetic activity of the extract and the presence of these compounds. This was confirmed by Pearson’s correlation coefficient, as shown in Table 1, revealing a strong correlation between the contents of total phenolic, total flavonoid, and total flavonol compounds and *α*-amylase inhibitory activity. Specifically, we found a significant positive correlation between TPC and *α*-amylase inhibitory activity (r = 0.6011). Moreover, a good correlation was observed between TFC and *α*-amylase inhibitory activity (r = 0.7891), while a strong correlation was found between TFL and *α*-amylase inhibitory activity (r = 0.8167). These findings collectively underscore the potential of Algerian *C. monogyna* as a therapeutic agent in the management of diabetes complications.

#### 3.4.3. Urease Inhibitory

**Figure 5:**
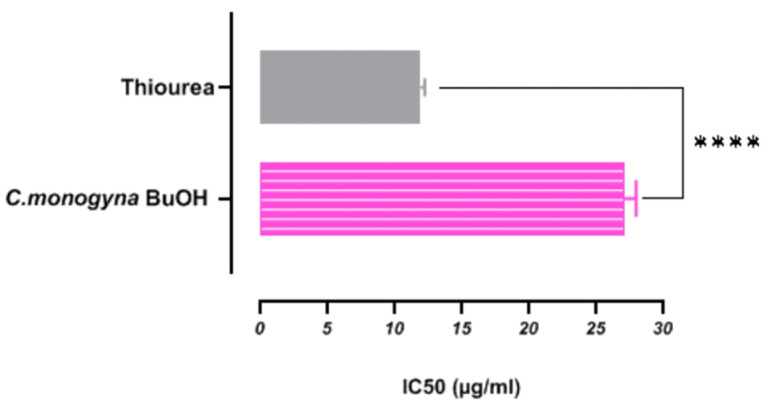
Urease inhibitory activity of butanolic fraction from *C. monogyna Jacq.* and **** indicate that significant at *p* value < 0.0001.

Recently, the study of the urease enzyme has become a vital area of research due to its broad implications for human health. Natural therapies have garnered significant attention, particularly concerning phytochemical compounds promising potent urease inhibitors. This enzyme is commonly found in various bacteria, fungi, algae, and plants and primarily catalyzes the hydrolysis of urea into ammonia and carbamate, representing the final step of nitrogen metabolism in living organisms [106]. The rapid and spontaneous decomposition of carbamate, catalyzed by urease activity, leads to excessive ammonia production. This process results in elevated body pH levels, which can have adverse effects on human health [107]. *Proteus mirabilis* and *Helicobacter pylori* typically exhibit higher urease activity than other microorganisms during human infections. This heightened enzyme activity is crucial for diagnostic, taxonomic, therapeutic, and vaccine development purposes in medical research [106]. In this regard, the inhibition of urease by the butanolic fraction of *C. monogyna* was investigated for the first time, as shown in Figure 5. Urease inhibition by the butanolic extract of *C. monogyna* exhibited significant inhibitory activity with an IC50 value of (26.36 ± 0.05 µg/mL) compared to the positive control thiourea (11.57 ± 0.10 µg/mL). To the best of our knowledge, no studies have previously reported the urease inhibitory effect of *C. monogyna* butanolic extract. The only study found on the effect of *C. monogyna* using a methanolic extract reported a lower inhibitory effect on urease, with an IC50 greater than 200 μg/mL [38], contrasting with our findings. The literature shows that polyphenols and flavonoids demonstrate notable urease activity, and our findings strongly support this data. Therefore, our findings provide a significant contribution to this field [106,107]. This is confirmed by the Pearson’s correlation coefficient shown in Table 1. We observed a very strong correlation between total phenolic, total flavonoid, and total flavonol content, and urease inhibitory activity (r = 0.9026, r = 0.9878, r = 0.9891), respectively. Polyphenols, including epicatechin, gallic acid, chlorogenic acid, and rutin, known for their antioxidant properties, may significantly contribute to the inhibition of urease activity [108]. This makes *C. monogyna* butanolic extract particularly valuable in developing treatments for conditions caused by urease-producing bacteria, such as urinary tract infections and peptic ulcers. Additionally, the presence of various polyphenolic compounds in *C. monogyna* may enhance its inhibitory effects, highlighting its potential as a promising natural alternative for managing urease-related conditions.

## 4. Conclusions

The study underscores the importance and effectiveness of the extraction methods used to obtain high levels of polyphenols, flavonoids, and flavonols in the butanolic extract of Algerian *C. monogyna*. Utilizing comprehensive LC-ESI-MS/MS analytical techniques, the study successfully detected and quantified high concentrations of phenolic molecules such as epicatechin, isoquercetrin, chlorogenic acid, quinic acid, rutin, hesperidin, astragalin, and nicotiflorin. This highlights *C. monogyna* as a valuable natural source of these biomolecules. The antioxidant activities of the lyophilized butanolic fraction were analyzed using five tests: DPPH, GOR, ABTS, CUPRAC, and reducing power assays. The extract showed highly effective antioxidant properties due to its rich polyphenolic content, making it suitable for neutralizing free radicals and preserving cells from oxidative stress. These properties are relevant to diseases such as diabetes mellitus, Alzheimer’s disease, and urease-related conditions. These findings suggest that Algerian *C. monogyna* holds potential as a therapeutic agent for managing diabetes complications and as a natural source of AChE inhibitors, making it promising for conditions related to urease activity. Its high concentrations of compounds with food preservation properties, such as epicatechin, chlorogenic acid, quinic acid, hesperidin, and astragalin, classify it as a plant rich in natural antioxidants. This makes it suitable for applications in medicine, pharmaceutical, cosmetic, and food sectors. It presents a natural alternative to synthetic antioxidants like BHA and BHT, avoiding their side effects and toxicity. The extract’s proven ability to extend food shelf life underscores its practical utility. These findings will stimulate future research and provide fundamental knowledge for exploring the isolation of specific components or developing novel herbal formulations from Algerian *C. monogyna* that are aimed at enhancing health benefits.

## Figures and Tables

**Table 1:** Pearson’s correlation values of TPC, TFC, and TFL contents and antioxidant (DPPH and ABTS) and enzymatic (ACHE, *α*-amylase, and Urease) activities of *C. monogyna* butanolic fraction.

IC50 TEST	Total Bioactive Component			Antioxidant Activity		Enzyme Inhibitory Activity		
	TFL	TPC	TFC	DPPH	ABTS	ACHE	*α*-Amylase	UREASE
TFL	1							
TPC	0.9342 ***	1						
TFC	0.9922 ***	0.9585 ***	1					
DPPH	0.9922 ***	0.9580 ***	1	1				
ABTS	0.7517 ***	0.5214 **	0.7201 ***	0.7201 ***	1			
ACHE	0.9885 ***	0.9701 ***	0.9989 ***	0.9990 ***	0.6902 ***	1		
*α*-amylase	0.8167 ***	0.6011 ***	0.7891 ***	0.8091 ***	0.9936 ***	NT	1	
UREASE	0.9891 ***	0.9026 ***	0.9878 ***	0.9787 ***	0.8134 ***	NT	NT	1

NT: not tested; **: indicate that significant at *p* < 0.01; and ***: indicate that significant at *p* < 0.001.

**Table 2:** The profile of various classes of phenolic compounds identified in the butanolic extract of *C. monogyna*.

N°	Analytes	Type of Compound	Quantitative Results (mg/g)
1	Quinic acid	Organic acid	37.819 ± 1.406
2	Fumaric acid	Dicarboxylic acid	0.540 ± 0.004
3	Aconitic acid	Tricarboxylic acid	N.D.
4	Gallic acid	Phenolic acid	0.249 ± 0.002
5	Epigallocatechin	Flavan-3-ols (catechin)	N.D.
6	Protocatechuic acid	Phenolic acid	0.210 ± 0.007
7	Catechin	Flavan-3-ols (catechin)	0.308 ± 0.006
8	Gentisic acid	Phenolic acid	0.069 ± 0.001
9	Chlorogenic acid	Phenolic acid (caffeic acid ester)	47.457 ± 1.010
10	Protocatechuic aldehyde	Phenolic aldehyde	N.D.
11	Tannic acid	Polyphenol (tannin)	N.D.
12	Epigallocatechin gallate	Flavan-3-ol (catechin gallate)	N.D.
13	Cynarin	Phenolic acid (caffeic acid ester)	N.D.
14	4-OH Benzoic acid	Phenolic acid	N.D.
15	Epicatechin	Flavan-3-ol (catechin)	99.916 ± 2.208
16	Vanilic acid	Phenolic acid	N.D.
17	Caffeic acid	Phenolic acid	N.D.
18	Syringic acid	Phenolic acid	N.D.
19	Vanillin	Phenolic aldehyde	0.133 ± 0.001
20	Syringic aldehyde	Phenolic aldehyde	N.D.
21	Daidzin	Isoflavone glycoside	N.D.
22	Epicatechin gallate	Flavan-3-ol (catechin gallate)	N.D.
23	Piceid	Stilbenoid glycoside	N.D.
24	p-Coumaric acid	Phenolic acid	N.D.
25	Ferulic acid-D3-IS	Phenolic acid (internal standard)	N.A.
26	Ferulic acid	Phenolic acid	N.D.
27	Sinapic acid	Phenolic acid	N.D.
28	Coumarin	Benzopyrone (coumarin)	N.D.
29	Salicylic acid	Phenolic acid	0.010 ± 0.0001
30	Cyranoside	Flavonoid glycoside (kaempferol-3-O-galactoside)	0.758 ± 0.027
31	Miquelianin	Flavonoid glycoside (quercetin 3-O-glucuronide)	0.013 ± 0.0002
32	Rutin-D3-IS	Flavonoid glycoside (internal standard)	N.A.
33	Rutin	Flavonoid glycoside (quercetin-3-O-rutinoside)	29.98 ± 0.740
34	Isoquercitrin	Flavonoid glycoside (quercetin-3-O-glucoside)	53.31 ± 1.172
35	Hesperidin	Flavanone glycoside	5.296 ± 0.177
36	o-Coumaric acid	Phenolic acid	N.D.
37	Genistin	Isoflavone glycoside	0.047 ± 0.0003
38	Rosmarinic acid	Polyphenol (caffeic acid ester)	0.035 ± 0.0004
39	Ellagic acid	Polyphenol (ellagitannin)	N.D.
40	Cosmosiin	Flavonoid glycoside (apigenin-7-O-glucoside)	0.994 ± 0.008
41	Quercitrin	Flavonoid glycoside (quercetin-3-O-rhamnoside)	N.D.
42	Astragalin	Flavonoid glycoside (kaempferol-3-O-glucoside)	1.774 ± 0.020
43	Nicotiflorin	Flavonoid glycoside (kaempferol-3-O-rutinoside)	1.482 ± 0.016
44	Fisetin	Flavonol	N.D.
45	Daidzein	Isoflavone	N.D.
46	Quercetin-D3-IS	Flavonoid (internal standard)	N.A.
47	Quercetin	Flavonoid (flavonol)	N.D.
48	Naringenin	Flavanone	N.D.
49	Hesperetin	Flavanone	N.D.
50	Luteolin	Flavone	N.D.
51	Genistein	Isoflavon glycoside	0.670 ± 0.022
52	Kaempferol	Flavonol	N.D.
53	Apigenin	Flavone	N.D.
54	Amentoflavone	Biflavonoid	N.D.
55	Chrysin	Flavone	0.043 ± 0.001
56	Acacetin	Flavone	0.063 ± 0.002

N.D.: not detected; N.A.: not applicable; and IS: internal standards.

**Table 3:** Antioxidant activities (μg/mL) of *C. monogyna* butanolic fraction.

Products	Radical Scavenging Activity IC50 (μg/mL)			Reducing Power A0.5 (μg/mL)	
	DPPH	ABTS	GOR	CUPRAC	Reducing Power
BuOH	16.80 ± 0.64 ^d^	4.98 ± 0.20 ^c^	3.82 ± 0.29 ^a^	10.93 ± 0.16 ^d^	14.78 ± 0.86 ^c^
Trolox *	5.12 ± 0.21 ^b^	3.21 ± 0.06 ^b^	4.31 ± 0.05 ^a^	8.69 ± 0.14 ^c^	5.25 ± 0.20 ^a^
BHT *	6.50 ± 0.58 ^c^	1.56 ± 0.24 ^a^	3.32 ± 0.18 ^a^	3.44 ± 0.04 ^b^	NT
BHA *	15.70 ± 0.41 ^d^	7.58 ± 0.69 ^d^	5.38 ±0.06 ^b^	1.34 ± 0.11 ^a^	NT
Ascorbic acid *	4.39 ± 0.01 ^a^	3.04 ± 0.05 ^b^	5.02 ± 0.01 ^b^	8.31 ± 0.15 ^c^	6.77 ± 1.15 ^b^
*α*-Tocopherol *	NT	NT	NT	NT	34.93 ± 2.38 ^d^

*: standard compounds. NT: not tested. Significant differences exist between the values with various letters (a–d) (*p* < 0.05).

**Table 4:** Acetylcholinesterase inhibitory activity IC50 values (µg/mL) for *C. monogyna* butanolic fraction and galantamine standard.

Extract	IC50 Values (µg/mL) for AChE Inhibitory Activity
BuOH	43.65 ± 2.10 ^b^
Galantamine	34.75 ± 1.99 ^a^

Significant differences exist between the values with various letters (a,b) (*p* < 0.05).

## Data Availability

The original contributions presented in the study are included in the article and supplementary material; further inquiries can be directed to the corresponding authors.

## Supplementary Materials

The following supporting information can be downloaded at: https://www.mdpi.com/article/10.3390/antiox13111350/s1, Figure S1: LC-ESI-MS/MS chromatogram of the standards. Table S1: Analytical method validation parameters for the LC-ESI-MS/MS method.
